# Preclinical profile of ITI-214, an inhibitor of phosphodiesterase 1, for enhancement of memory performance in rats

**DOI:** 10.1007/s00213-016-4346-2

**Published:** 2016-06-24

**Authors:** Gretchen L. Snyder, Jos Prickaerts, Marie-Louise Wadenberg, Lei Zhang, Hailin Zheng, Wei Yao, Sven Akkerman, Hongwen Zhu, Joseph P. Hendrick, Kimberly E. Vanover, Robert Davis, Peng Li, Sharon Mates, Lawrence P. Wennogle

**Affiliations:** 1 Intra-Cellular Therapies Inc., 430 East 29th Street, Suite 900, New York, NY 10016 USA; 2 Department of Psychiatry and Neuropsychology, School for Mental Health and Neuroscience, Maastricht University, P.O. Box 616, NL-6200 Maastricht, MD The Netherlands; 3 School of Natural Sciences, Linnaeus University, Kalmar, Sweden; 4 Tianjin Hospital, Tianjin, 300211 People’s Republic of China

**Keywords:** Novel object recognition, Phosphodiesterase-1, Cyclic GMP, Cyclic AMP, Memory, Conditioned avoidance response

## Abstract

**Rationale:**

Therapeutic agents for memory enhancement in psychiatric disorders, such as schizophrenia, are urgently needed.

**Objective:**

The aim of this study is to characterize the preclinical profile of ITI-214, a potent inhibitor of phosphodiesterase 1 (PDE1).

**Methods:**

ITI-214 was assayed for inhibition of PDE1 versus other PDE enzyme families using recombinant human PDE enzymes and for off-target binding to 70 substrates (General SEP II diversity panel; Caliper Life Sciences). Effects of ITI-214 (0.1–10 mg/kg, po) on memory performance were assayed in rats using the novel object recognition (NOR) paradigm, with drug given at specified time points prior to or following exposure to objects in an open field. ITI-214 was evaluated for potential drug-drug interaction with risperidone in rats using conditioned avoidance response (CAR) and pharmacokinetic assessments.

**Results:**

ITI-214 inhibited PDE1A (*K*_i_ = 33 pmol) with >1000-fold selectivity for the nearest other PDE family (PDE4D) and displayed minimal off-target binding interactions in a 70-substrate selectivity profile. By using specific timing of oral ITI-214 administration, it was demonstrated in the NOR that ITI-214 is able to enhance acquisition, consolidation, and retrieval memory processes. All memory effects were in the absence of effects on exploratory behavior. ITI-214 did not disrupt the risperidone pharmacokinetic profile or effects in CAR.

**Conclusions:**

ITI-214 improved the memory processes of acquisition, consolidation, and retrieval across a broad dose range (0.1–10 mg/kg, po) without disrupting the antipsychotic-like activity of a clinical antipsychotic medication, specifically risperidone. Clinical development of ITI-214 is currently in progress.

## Introduction

The cyclic nucleotide second messengers, cAMP and cGMP, have been well-characterized as intracellular signaling molecules in the brain where they have been implicated in the control of diverse processes including motor movement, learning, memory, and emotion. Cyclic nucleotide levels are determined by the balance between the adenylyl and guanylyl cyclases that catalyze their formation and the phosphodiesterase (PDE) enzymes that catalyze their hydrolysis and inactivation. The PDE superfamily is comprised of 11 families of PDE enzymes that are encoded by at least 21 distinct genes and classified based on substrate specificities (e.g., cAMP/cGMP) and regulatory factors (Conti and Beavo 2007; Francis et al. 2011). These enzymes display unique tissue distributions that differ among individual PDE families and structurally-related isoforms within the same enzyme family (Bender and Beavo 2006; Lakics et al. 2010).

Several PDE enzymes are expressed in abundance in the central nervous system (CNS) (Lakics et al. 2010). Certain of these enzymes are believed to modulate memory processes through regulation of cyclic nucleotide-dependent signaling pathways (Rutten et al. 2007; Reneerkens et al. 2009). Both cAMP and cGMP pathways, modulated by individual PDE families, have been shown to contribute to specific phases of memory (Izquierdo et al. 2002; Bollen et al. 2014; Rutten et al. 2007). Members of the PDE1 family are dual-substrate enzymes that hydrolyze and inactivate both cGMP and cAMP. Furthermore, within the PDE superfamily of enzymes, PDE1 is uniquely regulated by calcium/calmodulin-dependent signaling pathways (Bentley et al. 1992). Evidence suggests that PDE1 activity may be governed by phasic variations in calcium/calmodulin availability secondary to neuronal activity (Polli and Kincaid 1992; Polli and Kincaid 1994; Siuciak et al. 2007). There are three identified PDE1 isoforms (1A, 1B, and 1C) which are abundant in brain regions that subserve memory and learning, including frontal cortex, hippocampus, and striatum (Lakics et al. 2010). For example, the PDE1B isoform is highly enriched in neurons of the striatum and frontal cortex possessing cAMP signaling pathways controlled by dopamine D1-type receptors (Polli and Kincaid 1994; Lakics et al. 2010). Activation of dopamine D1 receptors on cortical neurons can enhance memory function (Castner et al. 2000) and, further, may be a plausible therapeutic approach toward overcoming cognitive impairment associated with schizophrenia and other CNS disorders (Castner et al. 2000; Goldman-Rakic et al. 2004).

Cognitive impairment is a core feature of psychiatric disorders such as schizophrenia that is largely unresponsive to antipsychotic medications (Tamminga et al. 1998; Hoff et al. 1999). Effective treatments for cognitive impairments in schizophrenia are needed as they can have a beneficial effect on functional outcome (Kahn and Keefe 2013). Short-term memory, which is relevant to memory acquisition processes (Akkerman et al. 2016), is in the domain of cognitive functions known to be impaired in schizophrenia (Targum and Keefe 2008). The novel object recognition (NOR) paradigm is useful for studying memory performance in rodents. This paradigm focuses, in particular, on memory processes such as acquisition, consolidation, and retrieval (Ennaceur and Delacour 1988; van Goethem et al. 2012). The latter two processes are required for long-term memory, which might be relevant for other psychiatric disorders, including Alzheimer’s disease. NOR is a valuable tool for identifying therapies preclinically which could enhance memory, although the clinical utility of such therapies must ultimately be evaluated in patients with schizophrenia, as some compounds (e.g., aripiprazole) have been falsely identified as beneficial for cognitive impairment, based on NOR results (Karamihalev et al. 2014; Nagai et al. 2009; Rajagopal et al. 2014). Of the various behavioral paradigms available for screening for potential cognitive improvement, NOR was selected given that effects of other PDE inhibitors have been well-characterized in this assay. In the context of cGMP-mediated effects versus cAMP-mediated effects on memory behavior by other selective PDE inhibitors, NOR could allow for characterization and interpretation of PDE1 inhibitors with dual substrate effects (Rutten et al. 2006; Rutten et al. 2007; Bollen et al. 2014).

An important requirement in developing therapies for treatment of cognitive impairment in patients with schizophrenia is the maintenance of efficacy of antipsychotic medications that are the standard of care for major psychotic symptoms of the disease. The conditioned avoidance response (CAR) assay is a well-characterized and widely-used rodent assay for screening compounds with antipsychotic-like activity (for review, see Wadenberg and Hicks 1999). The assay has predictive value for assessing antipsychotic-like activity since all clinically effective antipsychotic medications selectively suppress the CAR response (Arnt 1982; Wadenberg 2010). Used in concert with the NOR assay, CAR can be used to evaluate the potential of promising cognition enhancing therapies to improve memory performance without compromising the efficacy of a concurrent antipsychotic medication. Of note, no animal model of cognition, however, has translated into cognitive benefit for schizophrenia, to date.

Recently, we have discovered a portfolio of potent, selective, and brain permeant inhibitors of PDE1 (Li et al. 2016), enabling for the first time an examination of the role of PDE1 in memory function. One of these novel PDE1 inhibitors, ITI-214, is an investigational new drug that has recently been evaluated and found to be safe and well-tolerated in human clinical trials in healthy volunteers and patients with schizophrenia. Here, we present data from the preclinical characterization of ITI-214 in rats, indicating that the compound promotes improved memory performance in rats.

## Materials and methods

### Materials

ITI-214 (MW = 508) is (6a*R*,9a*S*)-2-(4-(6-fluoropyridin-2-yl)benzyl)-5-methyl-3-(phenylamino)-5,6a,7,8,9,9a-hexahydrocyclopenta-[4,5]imidazo[1,2-*a*]pyrazolo[4,3-*e*]pyrimidin-4-(2*H*)-one phosphate salt that was first synthesized by the medicinal chemistry department at Intra-Cellular Therapies Inc. (ITI). Risperidone and paliperidone (as reference compounds) were obtained from R&D Systems (Minneapolis, MN). Immobilized metal affinity fluorescence polarization (IMAP) reagents, including reaction buffer, binding buffer, Fl-cAMP, and IMAP beads, were from Molecular Devices (Sunnyvale, CA). FuGene6 transfection reagent and protease inhibitor cocktail tablets (Complete™, Mini, EDTA-free protease inhibitor cocktail tablets; Cat. No. 11 836 170 001) were from Roche Applied Science (Indianapolis, IN). All other chemicals were from Sigma-Aldrich (St. Louis, MO).

### Measurements of phosphodiesterase activity in vitro

Phosphodiesterases 1A, 1B, 1C, 2A, 3B, 4A, 5A, 7B, 8A, 9A, 10A, and 11A were generated from full-length human or bovine recombinant clones (designated below as r-xPDE). PDE6 was isolated from bovine retina.

#### PDE enzyme preparations

The following PDE enzymes were used: r-hPDE1A (HEK) (Accession No. NM_005019, *Homo sapiens* phosphodiesterase 1A, calmodulin-dependent, transcript variant 1); r-hPDE1B (HEK) (Accession No. NM_000924, *Homo sapiens* phosphodiesterase 1B, calmodulin-dependent, transcript variant 1); r-hPDE1C (HEK) (Accession No. NM_005020.1, *Homo sapiens* phosphodiesterase 1C, calmodulin-dependent); r-hPDE2A (Accession No. NM_002599, *Homo sapiens* phosphodiesterase 2A, cGMP-stimulated, transcript variant 1); r-hPDE3B (Accession No. NM_000922, *Homo sapiens* phosphodiesterase 3B) expressed in *Spodoptera frugiperda* insect cells (Sf9) using a baculovirus expression system, was from BPS Bioscience (San Diego CA, Cat. No. 60031); r-hPDE4A1A (Accession No. U97584, *Homo sapiens* phosphodiesterase 4A, transcript variant 1) expressed in Sf9 cells using a baculovirus expression system (BPS Bioscience, Cat. No. 60040); r-bPDE5A (Accession No. NM_174417, *Bos taurus* phosphodiesterase 5A) expressed in Sf9 cells; bPDE6 (from bovine retina rod) isolated from bovine retinas (Arvys Protein, Stamford, CT); r-hPDE7B (Accession No. NM_018945, *Homo sapiens* phosphodiesterase 7B) expressed via transient transfection of HEK293 cells; r-hPDE8A (Accession No. NM_002605, *Homo sapiens* phosphodiesterase 8A, transcript variant 1) expressed in Sf9 cells using a baculovirus expression system (BPS Bioscience, Cat. No. 60080); r-hPDE9A (Accession No. NM_002606, *Homo sapiens* phosphodiesterase 9A, transcript variant 1 was expressed via transient transfection of HEK293 cells; r-hPDE10A (Accession No. NM_006661, *Homo sapiens* phosphodiesterase 10A, transcript variant 2); and r-hPDE11A4 Accession No. BAB62712, *Homo sapiens* phosphodiesterase 11A, transcript variant 4) expressed in Sf9 cells using a baculovirus expression system was purchased from (BPS Bioscience, Cat. No. 60110).

#### Transient transfection

Transient transfection of HEK293 cells with recombinant protein expression vectors was performed using FuGENE 6 Transfection Reagent (Cat. No. 11 988 387 001, Roche Applied Science) according to the manufacturer’s recommendations. Mammalian expression cloning vectors with recombinant cDNA copies of each PDE gene were purchased from OriGene. Protein was expressed via transient transfection in HEK293 cells. Other PDE enzymes were expressed in Sf9 insect cells using the Bac-to-Bac baculoviral expression system (Invitrogen) according to the manufacturer’s instructions. After 48 h, cells were processed to obtain the soluble cytosolic fraction for assay.

#### PDE assays

PDE assays were performed in a reaction medium containing 10 mM Tris-HCl (pH 7.2), 10 mM MgCl_2_, 0.1 % BSA, and 45 nM Fl-cGMP or Fl-cAMP, respectively. IMAP assays were carried out for 15 min at room temperature and terminated by addition of binding reagent (Molecular Devices). Reaction mixture for assay of PDE1 activity also contained 30 μM CaCl_2_ and 10 U/ml calmodulin. The reaction mixture for assay of PDE2 contained 2 μM cGMP. Fluorescent-labeled cGMP (Fl-cGMP) was used as the substrate in the assays for PDE1, PDE5A, PDE6, and PDE9A, while fluorescent-labeled cAMP (Fl-cAMP) was used as the substrate for PDE2A, PDE3B, PDE4A, PDE7B, PDE8A, PDE10A, and PDE11A. Inhibitory concentration (IC_50_) values were calculated using nonlinear regression software, fitting a four-parameter one-site dose-response model (XLFit; IDBS, Cambridge, MA) and converted to *K*_i_ values using the Cheng-Prusoff equation (Cheng and Prusoff 1973).

### Off-target binding of ITI-214

The propensity of ITI-214 to bind biological target proteins unrelated to PDE family enzymes was evaluated by interrogating a panel of 70 key receptor, enzyme, and ion channel proteins comprising a diversity panel screen (General Side Effect Profile SEPII) performed by Caliper Life Sciences (now PerkinElmer). ITI-214 binding to each substrate was expressed as a mean percent of the reference control (*n* = 2 measurements) collected at a single, high (10 μM) concentration of drug.

### Treatment of animals for in vivo experiments

#### Rats

Male Sprague-Dawley rats (150–200 g), obtained from Charles River Laboratories (Wilmington, MA), were used for pharmacokinetic studies to evaluate possible drug-drug interactions with antipsychotic agents as performed at ITI using established protocols with this strain of rat. Wistar rats were used for all behavior studies, using established protocols in both the Prickaerts and Wadenberg labs. Male Wistar rats (280–350 g) were obtained from Charles River Laboratories (The Netherlands) for novel object recognition studies performed at The University of Maastricht (Maastricht, The Netherlands). Male Wistar rats (300–500 g) were used for tests of conditioned avoidance response in studies performed at Linnaeus University (Kalmar, Sweden). In all cases, animals were maintained in standard laboratory conditions under a 12-h light/dark cycle, except Maastricht with a reversed cycle, with food and water available ad libitum with a minimum of 1 week acclimation prior to experimentation. All animals were cared for in accordance with the Institute of Laboratory Animal Resources (1996), and all procedures were performed with the approval of the Institutional Animal Care and Use Committees at the respective institutions and contract research organizations.

#### Compound formulation for animal dosing

Unless otherwise indicated, ITI-214 was dissolved in a solution of 20 nM citrate buffer (pH 3.5) in 0.5 % (*w*/*v*) carboxymethylcellulose (CMC, 400–800 cP, #C4888; Sigma-Aldrich Chemical Co., Inc.) in water. The drug was administered, *per os* (po), by gavage to rats in a volume of 2 ml/kg body weight. Oral dosing solutions were prepared fresh daily. Risperidone was prepared for systemic dosing in the CAR studies by solubilization in a small volume of glacial acetic acid, which was further diluted with addition of a 5.5 % glucose solution (pH ~4.0). The pH of the dosing solution was adjusted to ~pH 5.5 by dropwise addition of 0.1 N NaOH in saline and volume adjusted by addition of saline in preparation for intraperitoneal (ip) dosing. For oral dosing, risperidone was suspended in a solution of 0.5 % CMC in water using bath sonication, and a suspension of drug was administered to rats via gavage (2 ml/kg body weight). Control treatments were always the vehicle solutions of the corresponding drugs.

### Measurement of memory performance using the novel object recognition paradigm

#### Object recognition memory

The object recognition test was performed as described elsewhere (Ennaceur and Delacour 1988; Akkerman et al. 2014). The apparatus consisted of a circular arena, 83 cm in diameter. Half of the 40-cm-high wall was made of gray polyvinyl chloride, the other half of transparent polyvinyl chloride. The light intensity was equal in the different parts of the apparatus, as fluorescent red tubes provided a constant illumination of about 20 lx on the floor of the apparatus. Two objects were placed in a symmetrical position at about 10 cm from the gray wall. Four different sets of objects were used. The different objects were (1) a cone consisting of a gray polyvinyl chloride base (maximal diameter 18 cm) with a collar on top made of brass (total height 16 cm), (2) a standard 1 L transparent glass bottle (diameter 10 cm, height 22 cm) filled with water, (3) a large metal cube (10.0 × 5.0 × 7.5 cm) with two holes (diameter 1.9 cm), and (4) a large aluminum cube with a tapering top (13.0 × 8.0 × 8.0 cm). The objects used in this study were carefully selected to be “novelty neutral,” that is to say the rats have no innate preference for one over the other. In all cases, objects were of sufficient size and weight that rats could not move the objects.

A testing session consisted of two trials. The duration of each trial was 3 min. During the first trial (T1), the apparatus contained two identical objects (samples). Rats were placed in the apparatus facing the wall at the middle of the front (transparent) segment. After the first exploration period, the rat was put back in its home cage. Subsequently, after a 24-h delay interval, the rat was put in the apparatus for the second trial (T2). The times spent in exploring each object during T1 and T2 were recorded manually using a personal computer.

Exploration was defined as follows: directing the nose to the object at a distance of no more than 2 cm and/or touching the object with the nose. Sitting on the object was not considered as exploratory behavior. In order to avoid the presence of olfactory cues, the objects were always thoroughly cleaned with a 70 % ethanol solution before each trial. All combinations and locations of objects were used in a balanced manner to reduce potential biases due to preferences for particular locations or objects.

#### Procedure

During the first 2 weeks after animal arrival, the animals were handled daily. They were then adapted to the procedure over the period of 2 days by allowing them to explore the apparatus (without any objects) for 3 min twice each day. The rats were considered to be adapted to the testing protocol when they showed a stable discrimination performance, i.e., a good discrimination after a 1-h interval and no discrimination after a 24-h interval (data not shown; for protocol, see Akkerman et al. 2012a). After this, the rats were assigned to treatment conditions in a balanced manner, thereby insuring an equal distribution of object combinations among the treatment conditions. In a first batch of 20 rats, drug was injected (0.1 mg/kg, 0.3 mg/kg, 1 mg/kg, and 3 mg/kg, given at 2 ml/kg po) either 2 h prior to T1 to test the dose response of ITI-214 for effects on memory acquisition or 2 h prior to T2 to test memory retrieval. Subsequent experiments with an additional cohort of 20 rats evaluated the effect of ITI-214 on early and late consolidation of memory by administering the drug at 4 min or 3 h after T1 (as described in Bollen et al. 2015). In all experiments, a 24-h inter-trial interval was used. In all cases, the experimenter was unaware of the treatment conditions that were being tested.

#### Statistical analysis

The basic measure recorded was the time spent by each rat exploring an object during T1 and T2. The time spent in exploring the two identical samples was represented by “a1” and “a2.” The time spent in T2 in exploring the sample and new object was represented by “a” and “b,” respectively. The following variables were calculated: e1 = a1 + a2, e2 = a + b, and d2 = (b − a) / e2. The e1 and e2 are measures of the total exploration time of both objects during T1 and T2, respectively. Differences in exploration times between treatment conditions were analyzed using a *t* test or one-way ANOVA. The d2 (discrimination index) is a relative measure of discrimination corrected for exploration activity (e2). Thus, the formula corrects for any differences between conditions in T2 exploration. One-sample *t* statistics were performed in order to assess per treatment condition whether d2 differed from zero (i.e., chance performance). However, comparison of the value of d2 with the value zero may not be the most suitable way for analyzing recognition (increased chance of making a type I error) (Akkerman et al. 2012b). Effects were, therefore, also assessed by a *t* test or one-way ANOVA. In case the ANOVA showed a significant difference between treatments, conditions were analyzed in more detail using post hoc Bonferroni *t* tests. A d2 of a treatment condition being different from zero as well as from its vehicle/control condition is considered to reflect a “full” effect, while only being different from zero is referred to as an “intermediate” effect (Akkerman et al. 2012b).

### Pharmacokinetic study of drug-drug interactions between ITI-214, risperidone, and its primary metabolite, paliperidone in rats

#### In-life procedures

Adult, male Sprague-Dawley rats (200–250 g in body weight; *n* = 3 rats/treatment condition) were administered vehicle (0.5 % CMC in water) or ITI-214 (3 mg/kg in vehicle) by gavage (po). All rats received a dose of risperidone (2 mg/kg, po) prepared as an even suspension in 0.5 % CMC. One hour later, the rats were killed and blood and brain tissue were collected. Brain tissue was transferred to Eppendorf tubes, then frozen at −80 °C until analysis. Blood, collected into tubes containing sodium citrate, was centrifuged (2000*g* × 15 min at 4 °C). Plasma was decanted into clean Eppendorf tubes and frozen at −80 °C until analysis. Levels of ITI-214, risperidone, and the major metabolite of risperidone, paliperidone, were quantitated in brain homogenates and in plasma, using standard reverse-phase high-pressure liquid chromatography with mass-spectrometric detection (HPLC/MS). Levels of each compound were expressed as ng/ml ± SD.

#### LC/MS analysis of drug in plasma and brain

Standard methods of tissue extraction were used. Whole brain was sonicated with 20 mM Tris-HCl, 135 mM NaCl, pH 7.4 buffer, giving a 200 mg/ml (*w*/*v*) homogenate. Brain homogenate or plasma was then extracted with two volumes of acetonitrile and clarified by centrifugation at 12,000*g* for 20 min. Extracts were separated by HPLC using a Waters Alliance 2695 separation module with a Sunfire™ C18 column (3.5 μm, 2.1 × 50 mm) and a gradient of methanol over 30 min in a mobile phase of 0.1 % formic acid with the separation monitored by LC-MS. Compound standardization was performed by methods analogous to those previously reported (Zhao et al. 2005; Appels et al. 2005). Brain and plasma concentration and ratio (brain/plasma) were calculated for each compound.

### Measurement of conditioned avoidance responding in rats

#### CAR apparatus and protocol

The behavioral procedures were conducted as described previously (Wadenberg et al 2007; Wiker et al. 2008). Briefly, adult male Wistar rats (*n* = 8) were trained and tested in a manually operated conventional shuttle box (530 × 250 × 225 mm; Kungsbacka Mät-och Reglerteknik, Kungsbacka, Sweden), divided into two equal compartments by a partition with an opening (75 × 75 mm). Upon presentation of an 80-dB white noise (White Noise Generator, USA), used as conditioned stimulus (CS), the rat had 10 s to avoid the unconditioned stimulus (UCS), an intermittent electric shock (~0.2 mA; with an inter-shock interval of 2.5 s, shock duration 0.5 s) in the grid floor, by moving into the opposite compartment. The shock was terminated within 50 s if rats failed to respond. Avoidance behavior, escape behavior, escape failures, and inter-trial crosses were recorded, as described here: (1) *avoidance* (response to CS within 10 s); (2) *escape* (response to CS + UCS); and (3) *escape failure* (if the rat was unable to respond to the shock by moving into the opposite compartment within 50 s, the trial was terminated). The inter-trial interval (end of trial to start of new trial) varied randomly between 20 and 40 s. Rats were trained for five consecutive days and adapted to the shuttle box 5 min before each training session. Training sessions consisted of about 20 trials, randomly distributed over 15 min. Only rats that performed at least 90 % avoidance on the last day of training were included in the experiments. All subsequent test sessions were preceded by a pre-test to verify continued performance in the paradigm. Pre-tests and test sessions consisted of approximately ten trials, each randomly distributed over a 7.5-min period. The same animals were tested repeatedly serving as their own controls (i.e., all rats received all treatments) in a change-over design (Li 1964). Experimental days were always separated by at least two non-experimental days. Experimental tests were separated by at least 2 days to insure drug washout.

#### Statistical analysis

Results from the conditioned avoidance responding (CAR) protocol were analyzed using non-parametric procedures. Statistical evaluation was performed by means of the Friedman two-way ANOVA by ranks followed by post hoc comparisons using the Wilcoxon matched-pairs signed-ranks test (Siegel and Castellan 1988).

## Results

ITI-214 is (6a*R*,9a*S*)-2-(4-(6-fluoropyridin-2-yl)benzyl)-5-methyl-3-(phenylamino)-5,6a,7,8,9,9a-hexahydrocyclopenta-[4,5]imidazo[1,2-*a*]pyrazolo[4,3-*e*]pyrimidin-4-(2*H*)-one phosphate salt (Li et al. 2016). The compound is a novel small-molecule investigational drug currently under human clinical evaluation as a medication for the treatment of psychiatric and neurodegenerative disorders.

### PDE potency and selectivity of ITI-214

ITI-214 was found to potently inhibit the activity of full-length recombinant r-hPDE1A (*K*_i_ = 34 pM), r-hPDE1B (*K*_i_ = 380 pM), and r-hPDE1C (*K*_i_ = 37 pM) enzymes transiently expressed in HEK cells. The compound expressed >1000-fold greater activity toward PDE1 isoforms compared with the next nearest PDE family enzyme, PDE4D (*K*_i_ = 33 nM) and 10,000–300,000-fold selectivity toward all other PDE enzyme families (Table 1).

**Table 1:** Inhibitory constant (*K*_i_) values for ITI-214 measured against a panel of phosphodiesterase isoforms. Values were determined as described in “Materials and methods” section using bovine or human recombinant enzymes, as indicated. Since for all PDE assays, substrate concentration was below *K*_m_ for the enzyme data are reported as *K*_i_ values. *K*_i_ values for ITI-214 against each PDE isoform were then expressed as a ratio over the *K*_i_ of the compound for human recombinant PDE1A enzyme

PDE target	K_i_ (μM)	Ratio PDEx/PDE1A
hPDE1A	0.000034	1
hPDE1B	0.00038	11.5
hPDE1C	0.000037	1.1
PDE1 (bovine brain)	0.000058	1.7
hPDE2A	>10	29,411
hPDE3B	3.1	93,939
hPDE4D	0.033	970
r-bovine PDE5A	0.63	19,090
bovine retina PDE6	0.32	9696
hPDE7B	0.36	10,909
hPDE8A	3	90,909
hPDE9A	>10	294,117
hPDE10A	1.8	54,545
hPDE11A	1.3	39,393

*hPDE1A* human recombinant PDE1A, *hPDE1B* human recombinant PDE1B, *hPDE1C* human recombinant PDE1C, *hPDE2A* human recombinant PDE2A, *hPDE3B* human recombinant PDE3B, *hPDE4A* human recombinant PDE4A, *r*-*bovine PDE5A* recombinant bovine PDE5A, *hPDE7B* human recombinant PDE7B, *hPDE8A* human recombinant PDE8A, *hPDE9A* human recombinant PDE9A, *hPDE10a* human recombinant PDE10A, *hPDE11A* human recombinant 11A

### Off-target selectivity of ITI-214

ITI-214 was assayed for potential off-target interactions against a standard panel of key proteins, including neurotransmitter receptors, ion channels, ion pumps, synthetic enzymes, and transporter proteins. This 70-target diversity panel [Side Effect Profile (SEP) II, Caliper Life Sciences], performed at a drug concentration of 10 μM, or roughly 300,000-fold in excess of the *K*_i_ of ITI-214 for inhibition of the PDE1A isoform, identified only 4 substrates showing >50 % binding to ITI-214, including Na^+^ channel (site 2), Ca^2+^ channel (L, DHP site), NK3, neurokinin receptor, and the dopamine transporter (Table 2).

**Table 2:** Off-target binding of ITI-214 to a 70-substrate side effect profile diversity panel (General SEP II) was conducted by Caliper Life Sciences (PerkinElmer) using a single drug concentration of 10 μM ITI-214, a concentration that is ~300,000-fold in excess of the *K*_i_ for PDE1 enzyme. A significant binding interaction in the assay was defined as >50 % binding to the target. The ten highest off-target binding interactions are shown in the table

Specificity profile for ITI-214—20 highest off-target interactions	
Target	% Binding
Na^+^ channel (site 2), sodium channel	88.44
Ca^2+^ channel (L, DHP site)	73.28
NK3 (h), neurokinin	64.65
DA transporter (h), dopamine	58.70
Alpha 1 (non-selective), adrenergic	40.90
Potassium channel I[Kr], (hERG)	35.09
Alpha 2 (non-selective), adrenergic	34.94
H3 (h), histamine	32.54
NK1 (h), neurokinin	30.93
B2 (H), bradykinin	30.79

*h* human, *DHP* dihydropyridine, *hERG* human ether-à-go-go-related gene

### The effect of ITI-214 on NOR in rats

**Fig. 1:**
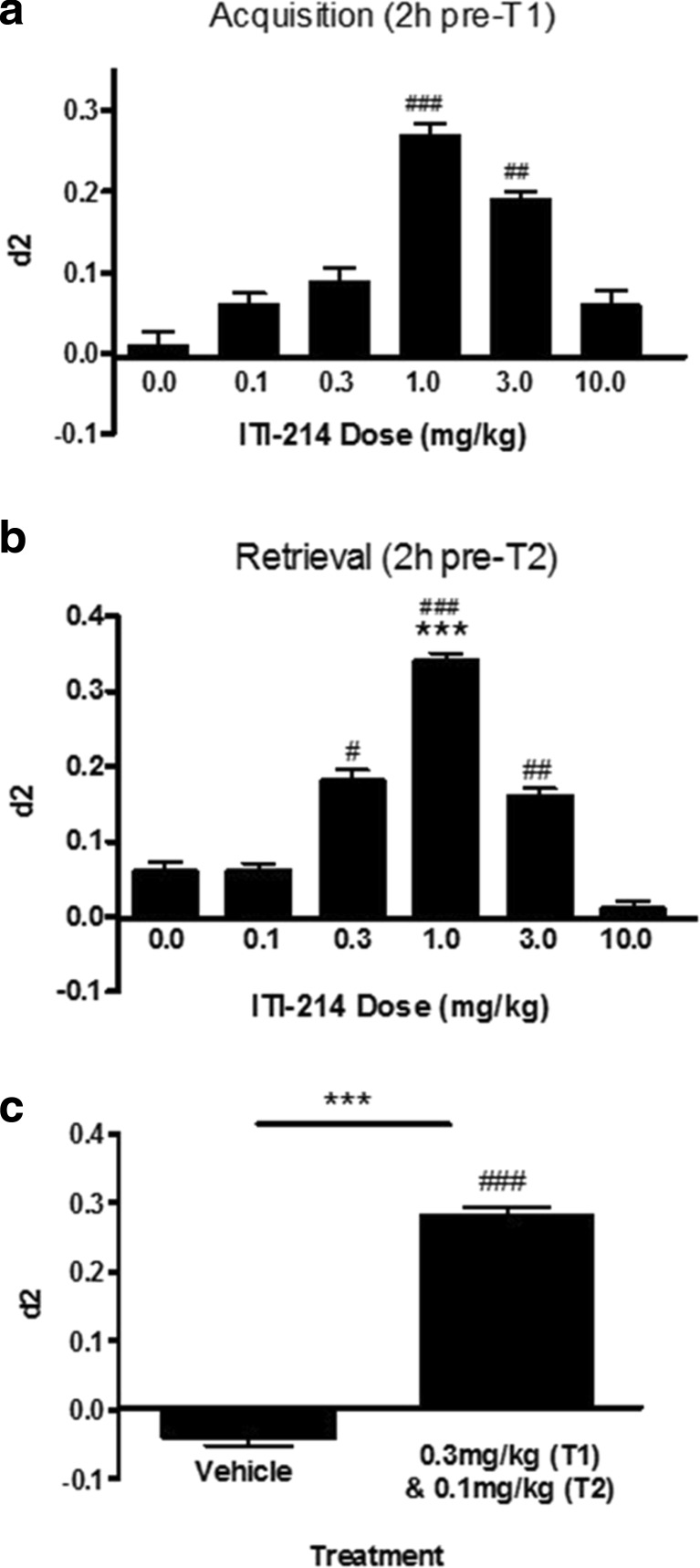
Synergistic effects of ITI-214 on memory performance in the novel object recognition paradigm in rats. Memory performance was measured in male Wistar rats using a 24-h interval. Rats were dosed orally with ITI-214 at the indicated doses and times relative to the T1 and T2 test periods to evaluate drug effect on memory acquisition (**a**) and retrieval (**b**) and to determine the optimal dose(s) of the compound for enhancement of memory performance. In **c**, a suboptimal dose of ITI-214 for memory acquisition (0.3 mg/kg, panel **a**) was administered to rats 2 h prior to T1 in combination with a suboptimal dose of ITI-214 for memory retrieval (0.1 mg/kg, panel **b**), which was administered 2 h prior to T2. Memory performance was then measured in T2. For all experiments, the discrimination index (d2) was calculated as a measure of time spent during T2 in contact with the novel object, compared with the familiar object. Discrimination index (d2) is expressed as mean ± SEM for *n* = 20 rats per experimental condition. The d2 measured after drug treatment was significantly different from zero (#*p* < 0.05; ##*p* < 0.01; ###*p* < 0.001, one-sample *t* test) and from vehicle condition [***p < 0.001, Bonferroni post hoc tests (**b**) or *t* test (**c**)]

Based on pharmacokinetic studies of ITI-214 in which a brain *T*_max_ at ~1.5 h was observed, we selected a 2-h drug pretreatment time for studies on memory processes and employed the NOR test with a 24-h interval between T1 and T2. To examine the dose response for ITI-214 effects on memory acquisition, the drug was administered to different groups of rats via gavage in a wide range of doses (0.1–10 mg/kg). The standard paradigm is to administer drug at 2 h prior to the T1, with effects on the d2 evaluated 24 h later during T2. ITI-214 treatment prior to T1 resulted in an increase in the rats’ ability to discriminate between objects at T2, i.e., d2 was different from chance (d2 = zero) performance with effective doses at 1 and 3 mg/kg (Fig. 1a). These were intermediate effects as the d2 measures for these two doses were not different from vehicle (*F*(5,18) = 2.09, n.s.). Under these conditions, ITI-214 had no effect on exploratory behavior measured during T1 or T2 (Table 3).

**Table 3:** Exploration times (in s) with administration of ITI-214 2 h before T1 in the NOR: acquisition. Different dosages of ITI-214 were administered 2 h before T1. The delay interval between the first and second trial was 24 h. *n* = 20 per experimental condition

Time PO	T1 −2 h Vehicle ITI-214	T1 −2 h 0.1 mg/kg ITI-214	T1 −2 h 0.3 mg/kg ITI-214	T1 −2 h 1 mg/kg ITI-214	T1 −2 h 3 mg/kg ITI-214	T1 −2 h 10 mg/kg ITI-214
e1	28.26 (2.19)	28.38 (1.86)	27.55 (1.62)	27.97 (1.78)	27.28 (1.74)	26.21 (1.97)
e2	25.68 (1.50)	27.33 (1.48)	26.10 (1.69)	29.38 (1.38)	28.73 (1.62)	28.15 (1.38)

No significant differences were found between treatment conditions in exploration times in T1 (e1: *F*(5,119) = 0.18, n.s.) and in T2 (e2: *F*(5,119) = 0.94, n.s.)

The effect of ITI-214 (0.1–10 mg/kg) on memory retrieval was evaluated by dosing the compound orally to rats 2 h prior to the T2 exploration session. ITI-214 resulted in significant increases in the d2 at doses ranging from 0.3 to 3 mg/kg, i.e., different from chance level, with a full effective dose at 1 mg/kg. This effective dose was statistically different from vehicle as well (*F*(5,119) = 4.65, *p* < 0.001; Fig. 1b). Given the effects of ITI-214 on both acquisition and retrieval processes, we examined the potential for synergistic effects of the sub-threshold doses of the drug on memory performance. For these studies, discriminatory behavior during T2 was measured in rats that had received a sub-threshold dose for memory acquisition (0.3 mg/kg) 2 h prior to T1 in conjunction with a sub-threshold dose (0.1 mg/kg) for effects on memory retrieval 2 h prior to T2. As shown in Fig. 1c, the combined dosing of ITI-214 at sub-effective doses prior to T1 and T2 resulted in a full increase in discrimination behavior, i.e., different from chance performance as well as vehicle treatment (*t*(38) = 4.35, *p* < 0.001). Importantly, ITI-214 had no effect on exploratory behavior in these experiments, whether tested during T1 or T2 periods (Tables 4 and 5).

**Table 4:** Exploration times (in s) with administration of ITI-214 2 h before T2 in the NOR: retrieval. Different dosages of ITI-214 were administered 2 h before T2. The delay interval between the first and second trial was 24 h. *n* = 20 per experimental condition

Time PO	T2 −2 h Vehicle ITI-214	T2 −2 h 0.1 mg/kg ITI-214	T2 −2 h 0.3 mg/kg ITI-214	T2 −2 h 1 mg/kg ITI-214	T2 −2 h 3 mg/kg ITI-214	T2 −2 h 10 mg/kg ITI-214
e1	26.48 (1.59)	29.32 (1.82)	28.83 (1.84)	26.81 (1.72)	27.20 (1.90)	30.59 (1.98)
e2	29.30 (2.19)	28.78 (2.08)	29.31 (1.52)	33.64 (2.35)	28.60 (1.13)	30.52 (1.87)

No differences were found between treatment conditions in exploration times in T1 (e1: *F*(5,119) = 0.81, n.s.) not in T2 (e2: *F*(5,119) = 0.99, n.s.)

**Table 5:** Exploration times (in s) with administration of suboptimal dosages of ITI-214 2 h before T1 and T2 in the NOR: acquisition and retrieval. Suboptimal dosages of ITI-214 were administered 2 h before T1 (0.3 mg/kg) and 2 h before T2 (0.1 mg/kg). The delay interval between the first and second trial was 24 h. *n* = 20 per experimental condition

Time PO	T1 −2 h / T2 −2 h Vehicle / Vehicle ITI-214	T1 −2 h / T2 −2 h 0.3 mg/kg / 0.1 mg/kg ITI-214
e1	31.90 (1.86)	30.43 (2.16)
e2	30.80 (1.43)	28.78 (1.26)

No differences were found between treatment conditions in exploration times in T1 (e1: *t*(38) = 0.52, n.s.) and T2 (e2: *t*(38) = 0.68, n.s.)

**Fig. 2:**
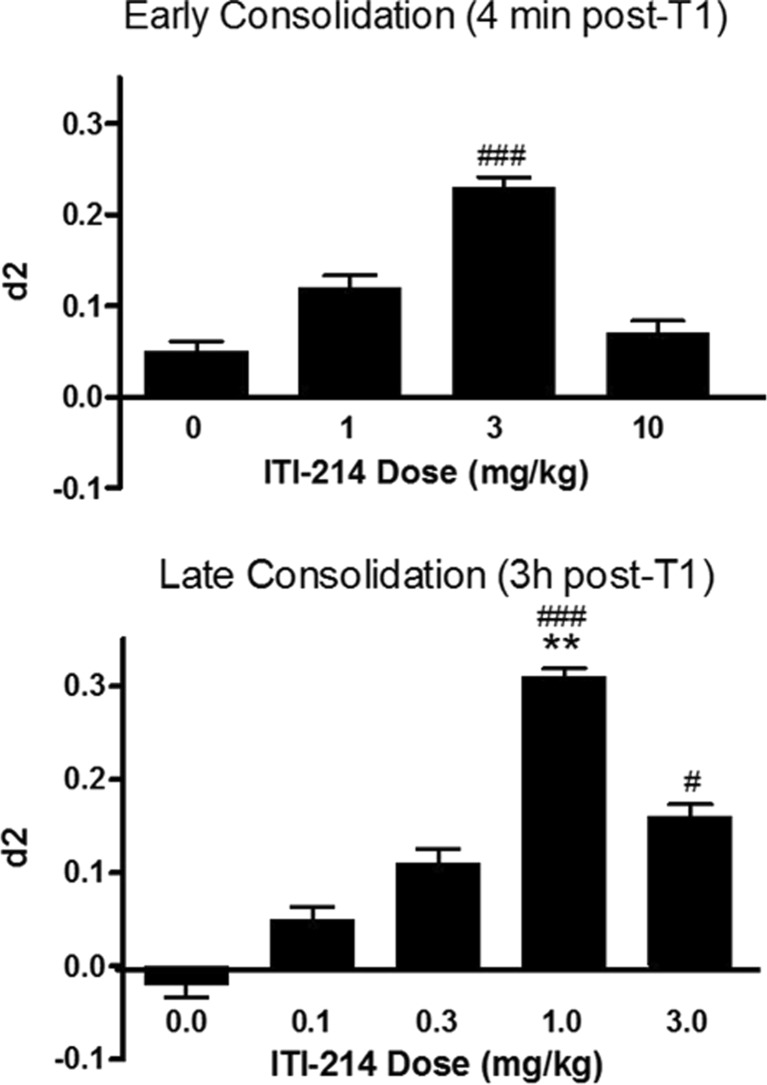
Effect of ITI-214, given after T1, on memory consolidation as measured in NOR. The effect of ITI-214 on different phases of memory consolidation was measured by dosing either 4 min after T1 (early consolidation, *top panel*; *n* = 20 per experimental condition) or 3 h after T1 (late consolidation; *bottom panel*; *n* = 12 per experimental condition). Discrimination index (d2) is expressed as mean ± SEM for *n* = 20 rats. The d2 measures after drug treatment were significantly different from zero (#*p* < 0.05; ###*p* < 0.001, one-sample *t* tests). Different from vehicle (**p < 0.01, Bonferroni post hoc tests)

The effects of ITI-214 on memory consolidation were examined by dosing the drug at specified times after the T1 exploratory session. To evaluate drug effects on the early consolidation of memory, ITI-214 was administered orally to groups of rats at a range of doses (1–10 mg/kg) shortly after the completion of the T1 period. ITI-214 administration resulted in an intermediate increase in d2, i.e., different from chance level, in animals that had received a 3 mg/kg dose within 4 min after T1 (Fig. 2, top panel). In this case, the d2 did not differ from vehicle (*F*(3,92) = 1.88, n.s.). Drug effects on the late phase of memory consolidation were studied by administering ITI-214 (0.1–3 mg/kg) 3 h after the T1 period. Twenty-one hours later, during T2, animals that had been treated with doses of 1 or 3 mg/kg ITI-214 displayed increases in d2 scores compared with chance performance and significantly different from vehicle (*F*(4,67) = 4.18, *p* < 0.01) with a full effective dose of 1 mg/kg. The results indicate improved late consolidation (Fig. 2, bottom panel). There were no effects on exploratory behavior in these consolidation experiments (Tables 6 and 7).

**Table 6:** Exploration times (in s) with administration of ITI-214 shortly after T1 in the NOR: early consolidation. Different dosages of ITI-214 were administered 4 min after T1. The delay interval between the first and second trial was 24 h. *n* = 20 per experimental condition

Time PO	T1 +4 min vehicle ITI-214	T2 +4 min 1 mg/kg ITI-214	T2 +4 min 3 mg/kg ITI-214	T2 +4 min 10 mg/kg ITI-214
e1	29.58 (2.83)	26.36 (1.93)	25.02 (2.20)	30.01 (1.62)
e2	25.56 (1.51)	29.66 (1.94)	30.55 (1.84)	31.35 (1.71)

No differences were found between treatment conditions in exploration times in T1 (e1: *F*(3,92) = 1.24, n.s.) and T2 (e2: *F*(3,92) = 2.15, n.s.)

**Table 7:** Exploration times (in s) with administration of ITI-214 3 h after T1 in the NOR: late consolidation. Different dosages of ITI-214 were administered 3 h after T1. The delay interval between the first and second trial was 24 h. *n* = 20 per experimental condition

Time PO	T1 +3 h vehicle ITI-214	T1 +3 h 0.1 mg/kg ITI-214	T1 +3 h 0.3 mg/kg ITI-214	T1 +3 h 1 mg/kg ITI-214	T1 +3 h 3 mg/kg ITI-214
e1	27.02 (1.71)	26.20 (1.95)	29.36 (3.22)	25.93 (1.53)	25.62 (1.89)
e2	30.97 (1.84)	32.81 (2.24)	29.64 (2.15)	27.86 (2.43)	28.34 (2.95)

No differences between treatment conditions in exploration times in T1 (e1: *F*(4,67) = 0.43, n.s.) and T2 (e2: *F*(4,67) = 0.68, n.s.) were found

We calculated the plasma concentration of ITI-214 associated with the highest dose of the drug (i.e., 3 mg/kg) having effects on memory measured 2 h after drug administration. The plasma level of the drug at this time was 30.1 ± 13.4 ng/ml (*N* = 12 rats) which corresponds to a free plasma concentration of 120 pM ITI-214. It should be noted that this free plasma level is close to the *K*_i_ values of the compound for inhibition of recombinant or bovine PDE1 enzymes (34–58 pM, Table 1) and well below the *K*_i_ of the compound for inhibition of the closest other PDE enzyme, PDE4D (33 nM, Table 1).

### Pharmacokinetic analysis of drug-drug interactions between ITI-214 and risperidone in rats

In order to evaluate the behavioral effects of ITI-214 achieved when dosed adjunctively with other psychiatric medications, we examined possible drug-drug interactions between the compound and the widely used antipsychotic medication, risperidone. Brain and plasma levels of risperidone and its active metabolite, paliperidone, were measured in samples from rats treated orally with risperidone (2 mg/kg, po) co-administered with vehicle solution (0.5 % CMC in water) or ITI-214 (3 mg/kg, po) in vehicle. One hour after drug treatment, risperidone, and the major metabolite, paliperidone, were abundant in rat brain homogenate and in plasma. Co-treatment of animals with ITI-214 had no significant effect on levels of risperidone in brain (22 ± 8 ng/ml in rats receiving vehicle and 16 ± 7 ng/ml in rats receiving ITI-214), or in plasma (85 ± 31 ng/ml in rats receiving vehicle and 65 ± 28 ng/ml in rats receiving ITI-214) (Table 8). Paliperidone was also detected in high levels in the brain (15 ± 4 ng/ml) and plasma (162 ± 70 ng/ml). In summary, co-administration of ITI-214 with risperidone did not significantly alter paliperidone levels in either rat brain (15 ± 5 ng/ml) or plasma (197 ± 91 ng/ml) (Table 8).

**Table 8:** Effect of co-administration to rats of ITI-214 and the antipsychotic drug, risperidone, on plasma and brain concentration of risperidone and paliperidone. Rats were given vehicle solution (0.5 % CMC in water) or ITI-214 (3 mg/kg, po) in vehicle. All rats received a dose of risperidone (2 mg/kg, po) prepared as an even suspension in 0.5 % CMC. One hour later, the rats were killed and blood and brain tissue collected for quantitation of levels of risperidone, and the major metabolite, paliperidone, in brain homogenate and in plasma, using LC/MS. Levels of both compounds are expressed as ng/ml ± SD (n = 3 animals/treatment condition)

Dosing condition	Risperidone (2 mg/kg)		Risperidone + ITI-214 (3 mg/kg)	
Analyte	Risperidone, ng/ml	Paliperidone, ng/ml	Risperidone, ng/ml	Paliperidone, ng/ml
Plasma	85 ± 31	162 ± 70	65 ± 28	197 ± 91
Brain	22 ± 8	15 ± 4	16 ± 7	15 ± 5

### The effect of ITI-214 on conditioned avoidance responding

**Fig. 3:**
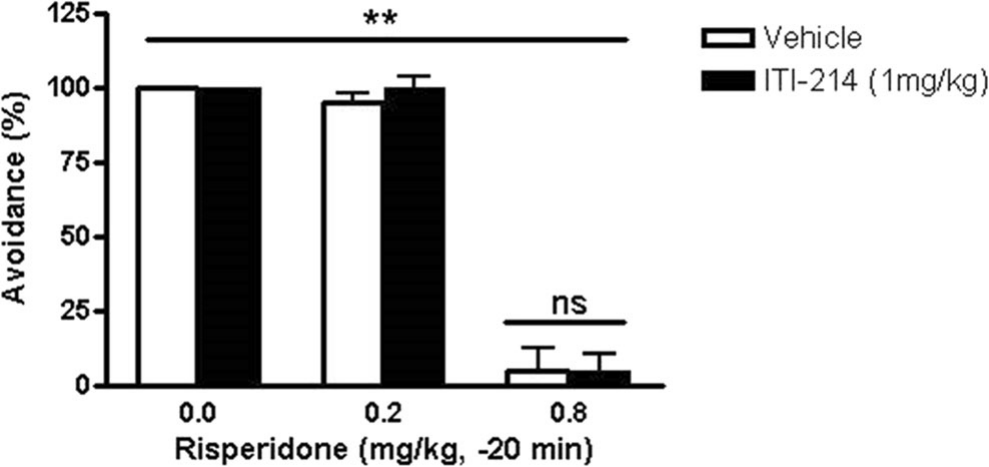
Effect of ITI-214 on performance of rats in the conditioned avoidance response (CAR) paradigm. The conditioned avoidance response was measured in rats treated with ITI-214 (1 mg/kg, po, −60 min) alone or in combination with the antipsychotic medication, risperidone (0.2 or 0.8 mg/kg ip, −20 min). Data are shown as medians ± semi-interquartile range based on repeated observations of the same eight animals rotated over all treatment groups serving as their own controls in a counterbalanced change-over design. **p < 0.001 compared with vehicle alone (Wilcoxon matched-pairs signed-ranks test); *ns* not significant compared with vehicle alone

Based on pharmacokinetic data indicating a lack of drug-drug metabolic interactions between ITI-214 and the antipsychotic drug (Table 8), risperidone, we next evaluated possible interactions between ITI-214 and risperidone on antipsychotic-like activity, as measured in the conditioned avoidance responding (CAR) paradigm in rats. Avoidance behavior was measured in rats after training in the CAR paradigm and tested after treatment with either a sub-threshold or supra-threshold dose of the antipsychotic medication, risperidone (0.2 or 0.8 mg/kg, i.p.), or vehicle solution (acidified saline). As anticipated, administration of the sub-threshold dose of risperidone (0.2 mg/kg) had no effect on the percent avoidances, whereas the higher, effective dose of risperidone (0.8 mg/kg) resulted in a significant inhibition of avoidance behaviors recorded (Fig. 3). When administered orally to rats, ITI-214 (1 mg/kg in 0.5 % CMC vehicle) alone had no significant effect on the percent avoidances. Furthermore, the same dose of ITI-214 did not affect avoidance behavior displayed by rats given the effective 0.8 mg/kg dose of risperidone (Fig. 3). Neither risperidone nor ITI-214 had significant effects on the number of escape failures in the CAR paradigm.

## Discussion

ITI-214 is an orally-bioavailable, small-molecule PDE1 inhibitor (Li et al. 2016) with picomolar affinity and high selectivity (1000-fold) versus other PDE enzymes. The PDE1 inhibitor displayed minimal off-target binding interactions, determined by surveying a broad range of 70 key targets represented in a standard panel of receptors, kinases, and enzymes linked to side effect liabilities.

Oral administration of ITI-214 was found to improve memory performance in rats, as reflected by effects on memory acquisition, consolidation, and retrieval. The effects of ITI-214 on memory were observed at dose levels that did not affect exploratory behavior or basal locomotor activity. Furthermore, these effects were expressed across a broad range of drug doses. The efficacy of ITI-214 in improving memory performance is consistent with the demonstrated role of both cAMP and cGMP signaling pathways in the circuitry of learning and memory (Kandel 2012; Prickaerts et al. 1997; Reneerkens et al. 2009). Several PDE enzymes have been shown to enhance memory performance in animals, including inhibitors of PDE5 (cGMP-specific) and PDE2 (both cAMP- and cGMP-specific) (Reneerkens et al. 2009). Another PDE enzyme implicated in memory is PDE4 (cAMP-specific), which has been demonstrated to modulate memory performance through genetic and pharmacological manipulations (Barad et al. 1998; Bourtchouladze et al. 1998). As shown here, ITI-214 is about 1000-fold more selective for inhibition of PDE1, compared with PDE4D. The optimal effects of ITI-214 on acquisition processes, shown here, were associated with plasma levels of ITI-214 corresponding to a brain free fraction estimated to be 120 pM ITI-214. This brain level of compound is close to the *K*_i_ of the drug for PDE1, but far below the *K*_i_ (38 nM) for inhibition of the closest PDE4 isoform, PDE4D. Although we cannot entirely discount possible influences of PDE4 enzyme inhibition in the observed memory effects of ITI-214, these data strongly support primary mediation of the effects through PDE1 inhibition.

The improvements in memory performance seen with ITI-214 are likely to reflect the substrate selectivity of the PDE1 enzyme family. As described by Izquierdo et al. (2002) and later by Rutten et al. (2007), cAMP and cGMP appear to subserve predominant roles in different phases of memory formation. PDE family enzymes that selectively regulate cAMP availability, like the PDE4 inhibitor rolipram, are specifically effective at promoting late consolidation (Rutten et al. 2007; Bollen et al. 2014). In contrast, inhibitors that promote cGMP, like the PDE5 inhibitor sildenafil, are most effective at enhancing early consolidation processes (Rutten et al. 2007; Bollen et al. 2014). PDE2A, an enzyme that controls both cAMP and cGMP levels, exerts effects on both acquisition and consolidation of memories (Rutten et al. 2007; Boess et al. 2004; Akkerman et al. 2015; Rutten et al. 2006). Taken together, we interpret the present data, demonstrating effects of ITI-214 across a wide dose range, as indicative of potent regulation by PDE1 of memory performance through actions on the acquisition, early and late consolidation, and retrieval of information. Of note, considering the *T*_max_ of ITI-214 (~1.5 h), the intermediate memory improvement observed when ITI-214 is given 4 min after T1 (early consolidation paradigm) might be explained, in part, by effects of the drug on late consolidation processes. That is to say, a 3 mg/kg dose of ITI-214 given immediately after T1 may deliver the dose level (1 mg/kg) necessary for a full effective late consolidation 3 h after T1.

The potent effects of ITI-214 on memory performance likely reflect the high abundance of PDE1 isoforms in brain neurons involved in learning and memory. PDE1A, 1B and 1C isoforms are abundantly expressed in frontal cortex, hippocampus, and striatum (Lakics et al. 2010). Although there is some debate about the exact roles for each brain region in the NOR, the hippocampus is considered as the site where object information from the perirhinal cortex is integrated with contextual information from the parahippocampal (or postrhinal in rat) cortex and temporal information from the frontal cortex, thus underlying the formation of a complete episodic memory (e.g., Winters and Bussey 2005; Aggleton and Brown 2006; Eichenbaum et al. 2007; Berkers et al. 2014). Though the present data are promising, it is clear that in order to have a view on the full potential of ITI-214 to treat cognitive deficits in schizophrenia and other disorders, the effects of the PDE1 inhibitor need to be investigated in other domains, including working memory per se, information processing, and attention, relevant to cognitive impairments specific for these disorders (for review, see Young et al. 2009). For example, follow-up studies to evaluate effects of ITI-214 on working memory in paradigms such as the radial arm maze (Young et al. 2009) or the delayed non-matching to position (DNMTP) tasks (Dudchenko et al. 2013), and those that report on attention and speed of information processing, such as the 5-choice serial reaction time test (Young et al. 2004), would be particularly informative. Testing of ITI-214 in paradigms for schizophrenia using pharmacologically induced deficits (e.g., chronic PCP or MK-801) would also be very useful (e.g., see Rajagopal et al. 2014). Many of the aforementioned tasks are represented in the Cambridge Automated Neuropsychological Testing Assay Battery (CANTAB) which could be used in future studies to translate the results with ITI-214 in rodent paradigms into non-human primate and human patient populations (Young et al. 2009; Young and Geyer 2015).

The antipsychotic-like activity of risperidone, as measured by blockade of conditioned avoidance responding (CAR), was found to be equivalent in the absence or presence of an optimal memory-enhancing dose of ITI-214. Co-administration of this dose of ITI-214 with risperidone had no significant effect on the brain and plasma level of parent drug or its major active metabolite, paliperidone. The lack of effect of the ITI-214 on antipsychotic-like activity of risperidone and the absence of significant drug-drug interaction with this antipsychotic medication supports its potential as an adjunctive treatment for schizophrenia.

In conclusion, cognitive impairment is widely accepted as a core feature of schizophrenia (Tamminga et al. 1998; Hoff et al. 1999; Lieberman et al. 2005). Patients with first-episode schizophrenia score one to two standard deviations below healthy control subjects on cognitive tasks (Hoff et al. 1999). Residual symptoms including negative symptoms, depression, and cognitive impairment substantially contribute to the diminished quality of life of patients with schizophrenia (Ueoka et al. 2011) even after the acute hallucinations and delusions are controlled. Current antipsychotic medications do not effectively improve, and often worsen cognition in patients (Cancelli et al. 2009). The present study provides preclinical data supporting the efficacy of a potent and selective PDE1 inhibitor, ITI-214, for enhancement of memory performance in a manner which does not significantly alter antipsychotic activity of a widely used antipsychotic medication, risperidone. Recently, ITI-214 has been found to be safe and well-tolerated in phase I human clinical safety evaluations in healthy volunteers and patients with schizophrenia. The potential of PDE1 inhibitors like ITI-214 for improvement of cognitive performance in patients with neuropsychiatric and neurological indications will require further validation as the agent undergoes comprehensive human clinical evaluation.
